# The Seventy-second Anniversary of the Medical Society of London

**Published:** 1845-06

**Authors:** 


*The Seventy-second Anniversary of the Medical Society of London* was held on the 6th of March, at the London Coffee House; the President, Dr. Theophilus Thompson, in the chair. The Fothergillian Gold Medal was presented to Walter C. Dendy, Esq., for an Essay on Lepra and Psoriasis; and the Silver Medal of the Society to Dr. Chowne. Dr. Marshall Hall delivered the Oration—On the influence of the Mind on the Body. The ballot was declared as follows:—President: Dr. T. Thompson. Vice-Presidents: Dr. M. Hall, Mr. Pilcher, Dr. Golding Bird, Mr. Headland. Treasurer: Mr. Clifton. Librarian: Mr. Dendy. Secretaries: Mr. W. A. Harrison, Mr. J. F. Clarke. Secretary for Foreign Correspondence: Dr. Davidson.—*London Lancet.*

